# Protective strategies and motivations to control drinking among Brazilian college students: a qualitative study

**DOI:** 10.1186/s12889-023-16854-7

**Published:** 2023-12-01

**Authors:** Marina Noto Faria, Elaine Lucas dos Santos, Ana Regina Noto, André Bedendo, Maria Lucia O. Souza-Formigoni

**Affiliations:** 1 Department of Psychobiology, Universidade Federal de São Paulo, São Paulo, Brazil; 2 Biological Sciences Center, Universidade Estadual do Norte do Paraná- UENP, Campus Luiz Meneghel, Jacarezinho, Brazil; 3 Department of Health Sciences, University of York, York, UK

**Keywords:** College Students, Protective behavioral strategies, Alcohol consumption, Self-care, Motivations

## Abstract

**Background:**

To develop prevention programs or early interventions to reduce alcohol consumption and related problems among college students, it is essential to understand their motivations for drinking and the spontaneous (effective and non-effective) strategies they employ to control, considering the social and cultural contexts influence. This study aimed to explore these factors and the student’s application of selfcare in different situations and environments, as well as to identify their reasons for not drinking.

**Methods:**

The students were invited to participate using a snowball sampling, up to the theoretical saturation point. Qualitative individual semi-structured interviews were carried out and the interviews contents were analyzed using the NVivo software. The participants were 23 college students between 18 and 24 years old, with diverse patterns of alcohol use (low-risk to suggestive of dependence).

**Results:**

Data analysis highlighted three main themes: (a) Contexts (such as bars, “open bar” parties and others) and consumption patterns; (b) Protective Strategies (such as stop-drinking intervals, eating before or during drinking, returning home in the company of a friend); (c) Motivations to control drinking (such as sense of responsibility, bad previous experiences, family and religious issues). Protective strategies and motivation to control drinking were perceived to be less prominent in specific contexts that favor high alcohol consumption, as open bar parties.

**Conclusions:**

Motivations and protective strategies varied according to the drinkers’ profile, social situations and settings in which they consumed alcohol. The results highlight the need for preventive interventions planned for specific drinking patterns and contexts.

## Background

Alcohol consumption among young people is a behavior that poses several risks to their safety. Even though alcohol use in Brazil is forbidden for those under 18 years of age, 34.3% of adolescents from 12 to 17 years old reported having drunk alcohol in the last twelve months, according to the 3rd National Survey on Drug Use by the Brazilian population [1]. Moreover, 8.8% of those surveyed had been drinking in a binge pattern, defined as the intake of at least four standard drinks (for females) or five (for males), corresponding to 48 and 60 g of pure alcohol, respectively, in a two-hour period. The same survey showed a high prevalence of alcohol consumption in those aged between 18 and 34 in the last 12 months (72.1% and 74.5% among 18–24 and 25–34 years old, respectively). The prevalence of binge drinking was 35.1% and 38.2% among 18–24 and 25–34 years old, respectively. Considering the educational background, a higher binge drinking prevalence was observed among those with complete university level (43.9%) than in those with complete high school or incomplete university levels (35.7%).

A previous study in São Paulo State (Brazil) with college students [2] reported that 21.8% of the participants were classified as “at-risk” or “suggestive of dependence” users, according to the scores of the Alcohol, Smoking and Substance Involvement Screening Test (ASSIST) [3].

Eckschmidt et al. (2013) [4] compared the alcohol consumption of Brazilian and North American college students and found similarly high rates (respectively 88.6 and 89.3%) of lifetime alcohol use. Other authors also found high-risk drinking rates in young adults in the United States [5]. Among the short-term consequences of alcohol use in youth, there are many risk behaviors, such as being a passenger in a vehicle driven by someone who has consumed alcohol; acting impulsively and then regretting it; having unprotected sex; drink driving; having problems with close relationships; being involved in car accidents; having problems with the law, skipping classes and hurting themselves or others [6]. Binge drinking is widely associated with a state of intoxication that leads to risky behaviors and consequences such as hangovers, blackouts, memory loss, nausea and vomiting [7]. Blackout behavior is considered a marker of problematic alcohol consumption [7]. Approximately 50% of young adults who drink reported that they blacked out at least once in their life [8]. This phenomenon occurs when an individual reaches a high level of alcohol intoxication, which can damage the cellular communication of the hippocampus, and other structures and regions related to it, resulting in partial or complete memory loss [9, 10]. A qualitative study carried out in the USA analyzed the knowledge of college students about the risk factors associated with alcohol-induced blackout. Participants reported as the main factors that increased its likelihood: heavy drinking in a short period of time; drinking on an empty stomach; and drinking alcohol in combination with caffeine, antibiotics, cough medicine, painkillers, Xanax, and cocaine. Some of the students reported having alcohol tolerance is a protective factor for blackout [11].

Protective behavioral strategies (PBS) are cognitive and behavioral strategies aimed at decreasing heavy alcohol use and alcohol problems. The most common types of strategies include limiting or stopping drinking, drinking control or adopting harm reduction [12–14]. PBS have been considered important tools in preventive programs. Some recent studies suggested that the use of PBS by young people are greatly influenced by the drinkers’ profile and the context of use [14]. The motivations for drinking seem to influence the probability of drinkers using these strategies, either facilitating or hindering them [15]. Regarding the context of use, despite the large literature on PBS, little is known about how young people choose to adopt PBS in specific social contexts [16].

Motivations can be defined as reasons, conscious or not, for behaving in a certain way [17]. Studies have shown that the motivations for alcohol consumption are strongly related to the context in which it occurs, a reason why it is important to understand the phenomenon among college students [18, 19] as it can affect, in different ways, the use and patterns of consumption [20].

There are different theoretical models that aim to clarify how behaviors are expressed in social contexts. The influences of psychosocial and cognitive-behavioral parameters can be interpreted from the perspective of the social norms theory [21, 22], which stresses the importance of the influence of relational aspects for understanding human motivations and behaviors. However, individual capacity for observation and self-regulation of consumption also seems to be essential [23–25]. The social norms theory posits that social behavior stems from learning by means of operant conditioning and imitation, with individuals tending to behave according to the norm, that is, they generally adjust their behavior to be close to what the group they belong to considers “normal”. However, students often have misperceptions about the normative behaviors of risk and protection, which leads to a distorted adjustment of behavior [21, 22].

In addition to the normative social context, alcohol use among college students is influenced by motivations that can be external or internal [18]. External motivations are associated with family, friends and the media - such as alcohol advertisements. Internal motivations are related to entertainment, curiosity, low self-esteem, the need to belong and a wish to forget problems [18]. Motivations not to drink are resources that strengthen self-regulation, influencing and reinforcing the behavior. The relationship between alcohol consumption and self-regulation has, therefore, been the focus of several studies [23–25]. Self-regulation is the capacity to control one’s immediate desires to gain future benefits. Motivations for the control of consumption strengthen the self-regulation of students when facing a situation where there are alcoholic beverages. Therefore, they might be considered an important part of self-care [26, 27] that is, a set of daily behaviors for the prevention of illnesses and the maintenance of well-being [28]. Although the literature contains many studies that describe the motivations for the consumption of alcohol [29–31], few studies have evaluated the motivations for drinking control or strategies of personal care to minimize risks among college students in low- or middle-income countries. Most of the studies in this area employed epidemiological methodologies and questionnaires with closed questions, which limit the types of possible answers. According to Merrill et al. (2021) [32], qualitative methods can elucidate why certain drinking behaviors occur from a drinker’s perspective, helping to understand the associated internal and external factors. These authors, as have many others, studied the predictors of high intensity drinking (HID) episodes in samples of individuals with regular high alcohol consumption, classified either as at-risk of dependence or already dependent. However, there is a paucity of data in the literature on internal and external factors associated with low drinking levels, as well as on the strategies used by young adults to cope with drinking situations and avoid the related problems, such as blackout behavior.

A recent study by Stevens et al. (2021) [33] pointed out that although there are many studies focused on the motivations related to why individuals drink, studies on reasons for not drinking at all or on a given day are less frequent. They reported that the variability in non-use of alcohol on days when the use was planned occurred more frequently at the within-person level (26%) than at the between-person level (74%). They recommended future studies could benefit from offering a free response option to better explore these issues. This kind of study could make a significant contribution to enhance alcohol or other drugs-related preventive or harm reduction interventions.

Given these gaps in the literature, this study used a qualitative approach to analyze Brazilian college students’ protective behavioral strategies and motivations to control drinking, taking into account internal and external factors associated with alcohol consumption, in a sample of urban college students with “low” or “at risk” levels of alcohol consumption in diverse drinking settings.

## Methods

Qualitative research procedures were used for this study to describe and analyze college students’ beliefs and behaviors about alcohol consumption. Content analysis allows evaluating the phenomenon using the subjects’ conceptual framework [34, 35].

### Participants

An intentional criterion sampling was used for selecting the participants, who were 23 college students over 18 years old. The exclusion criteria were evident cognitive impairment or psychiatric disorder that could bias the interview. They were enrolled in universities in the city of São Paulo (N = 18) and the surrounding area (N = 5) and a criteria for inclusion was to had consumed alcoholic beverages, at least occasionally. Convenience sampling and snowball sampling techniques were used to include participants. This sampling technique consists of creating a chain of new participants starting from the first respondent who, in turn, suggests another college student to be interviewed [36]. The first participants of each chain were invited through social media (Instagram or Facebook). A maximum of three participants per network were invited to the study. None of the college students interviewed refused to participate or dropped out. The data collection was completed when the contents of interviews became redundant, indicating the “point of theoretical saturation” was achieved. This occurs when significant new information was no longer obtained regarding the contexts of alcohol consumption, motivations to drink and to stop drinking, and protective behavioral strategies (PBS) [36, 37]. The authors determined this point using a triangulation process, focusing the PBS mentioned by the students regarding different patterns and contexts of alcohol use.

### Interviews

Semi-structured individual interviews took 24 min on average and started after had granted permission for the interview to be recorded. All interviews were carried out by the first author, a psychology student, trained and supervised by researchers with extensive experience in qualitative studies. All the interviews were conducted in public environments with little movement or noise, in coffee shops or isolated places on the university campus. The interviews were carried out before the COVID-19 pandemic, between August 2019 and February 2020. After the interviews, the first author completed the field diaries with comments about the participants’ behaviors and interview process. The interviews were audio-recorded for the subsequent transcription of their full content. The transcripts were not returned to participants for comment or correction. The interviews followed a semi-structured script which included open questions about the contexts of alcoholic beverage consumption, motivations to drink or not and protective behavioral strategies used by the participants. Cognitive-behavioral approaches and the theory of social norms were used to design the interview script [15, 18, 21, 23, 25]. The interviews began with generic questions to establish rapport between interviewer and interviewee, followed by the main questions: “*How do you perceive alcohol consumption among college students?*”, “*In what contexts does consumption occur?*”, “How does consumption occur?”, “How do you realize that you’ve crossed the line?”, “What are the motivations for not crossing the line?”, “What measures of self-care do you take (protective behavioral strategies)?”.

Although the participants had reported their alcohol consumption during the interview, to classify their pattern of use, after the interview we asked them to complete the Brazilian version of the Alcohol Use Disorders Identification Test (AUDIT) [38]. The AUDIT has been used for the identification of at-risk drinkers among college students [39], including questions about frequency of drinking, drinks consumed on a typical day, frequency of heavy drinking and alcohol-related problems. According to their AUDIT scores, the participants were classified into four zones: *low-risk* (< 8), *hazardous use* (8–15), *harmful use* (16–19) or *suggestive of dependence* (> 19).

### Processing and content analysis

All stages of data collection and analysis were triangulated and supervised. In the data collection stage the authors analyzed the data in depth to create categories and determine the theoretical saturation point [40] The categories were based on the participants’ discourse, field diary and qualitative information available in the literature on alcohol consumption and motivation to drink. The main categories were: *Self-care strategies, Group dynamics and Memories of personal experiences*. All the data were fully transcribed and inserted in NVivo 12 software [41], to organize and perform the analysis. The data analysis process consisted of six steps: skimming the first interviews, creating initial codes, skimming other interviews, reviewing initial codes, defining main and final categories. Skimming the interviews consists of quickly reading the transcriptions, identifying the themes that emerge from the participants’ discourse [34, 35]. In order to guarantee the quality of the categorization, a triangulation process was performed by four researchers: three Ph.D. psychologists (ARN, AB, ELS) and one psychology student (MNF).

In order to ensure the confidentiality of the information provided by the participants, an alphanumeric code was defined to identify the interviews using: *interview number (order of interviews), the gender of the respondent (“F” - female; “M” - male), the stage of the college course they are in (“F” - first half of the course; “S” - second half of the course), the general area of the course (“H” - humanities, “HL” - health, “E” – exact sciences) and the educational institution (“1” – public, “2” – private). Therefore, a participant with the code “06MSH1” would mean: sixth interview (06), male respondent (M) in the second half of the course (S), main area Humanities (H) at a public institution* [1].

#### Ethical aspects

Before starting the interview, all participants signed an informed consent form and had the opportunity to ask for any additional information if they had doubts about the research project. They were informed about their right to request partial or final data of the research as well as the exclusion of their data from the research at any stage of the project. The study was submitted to and approved by the UNIFESP Research Ethics Committee (Report 2.450.631/2018).

## Results

### Profile of the participants

The participants were between 18 and 24 years old, with an average age (mean ± SD) of 20.2 ± 1.6 (women) and 21.6 ± 1.7 years (men). Out of the 23 students interviewed, eleven (47.8%) were female. Most of the students (78.2%) were from private institutions, enrolled in Humanities courses (66.8%). The others were in the same proportion from Health (16.6%) and Exact Sciences (16.6%) areas. Most of them (60.8%) were in the second half of the course. As regards alcohol consumption and related problems, their average AUDIT scores were in the beginning of the hazardous use zone (M: 8.6, SD: 7.0) and women presented lower means (M: 6.9, SD:5.4) than men (M: 10, SD:8). Among the 23 participants, just one woman but no men reported not having been using alcohol recently. Most of the participants, five women (45.5%) and seven men (53.8%) were classified in the AUDIT *low-risk* zone; four women (36.4%) and three (23.1%) men in the *hazardous use zone;* one woman (9%) and no man in the *harmful use* zone, and no woman and three men (23.1%) in the *suggestive of dependence* zone (AUDIT scores > 19).

The participants answers to the questions about their perception of alcohol consumption among college students, their motivations for controlling it and the contexts where they drink were submitted to a qualitative analysis. We identified four main themes: “*Contexts and alcohol consumption patterns”*; “*Protective strategies”; “Motivations for controlling alcohol consumption” and “Influence of patterns of alcohol use and the environment”.* All participants reported having already attended environments where alcohol consumption was available.

### Contexts and alcohol consumption patterns

When participants were asked about the contexts in which their alcohol consumption happened, most of them associated the pattern of use with the environments where it is consumed, as well as with the people present in the specific place and the kind of social interaction at the time of consumption (Table 1**)**. The main places of use they mentioned were bars, “open bar” parties (admission fee includes free drink consumption), cash-bar parties (pay for each drink), nightclubs, living spaces in the university, meetings at fraternities or friends’ houses, and in street environments (including street meeting points and consumption in front of convenience stores or bars).

**Table 1:** Examples of sentences on “Contexts and drinking patterns” reported by the participants

Categories	Examples
Bar	*“You know, in the bar people are more interested in talking. I don’t think people drink that much (…) I feel that people drink smaller amounts throughout the evening (…)” (06MSH1-R)*
Open bar parties	*“(…) I think, at the parties (open bar parties) alcohol consumption is always heavier than in other places (…) because you’re in an environment that encourages you to drink (…) “drink, drink, drink, drink” all the time (…)” (11MSH1-SD).* *“(…) you know, it’s an open bar. Imagine you are at a party, you’ve already paid to be in there and you can get “free” drinks. It’s inviting you to drink (…).“(07MSH1-SD).*
Cash-bar parties and nightclubs	*“(…) but I think at the cash-bar parties (…) the consumption is lower, right?(…) Because you have to buy your drink all the time, you know! (…)”. (02MSH2-LR)*
Living spaces in the university	*“(…)For example, after the week is over, on a Friday, people gather somewhere in campus (…) sometimes drinking a beer or two (…) and I don’t see it as a problem. But sometimes people end up exaggerating.” (02MFH2-LR)*
Meetings at friends’ or fraternities	*“I think (…) depending on the weekend, people usually get together with their friends, open a beer, drink a gin and tonic.” (*15MSH *-R)* *“(people drink) In a college fraternity or sometimes at home(…) people get sick and blackout. But, in general, this mostly happens at parties (the highest consumption).”* (21MSHL2-LR)

Note: In order to maintain the confidentiality of the participants’ data, we created an alphanumeric code to identify the interviews, following the sequence: interview number (order of interviews), the gender of the respondent (“F” - female; “M” - male); the phase of college course they are in (“F” - first half of the course; “S” - second half of course), the general area of the course (“H” - humanities, “HL” - health, “E” – exact sciences) and the type of educational institution (“1” – public, “2” – private and the last part (after the hyphen) refers to their classification according to the AUDIT into: LR-Low risk, R- Risk or SD- Suggestive of dependence)

The participants considered bars as a relatively controlled consumption environment, used with the main purpose of socializing through conversations. On the other hand, “open bar” parties were reported as an environment of more intense consumption. Participants recognized in these places the presence of many environmental triggers for alcohol consumption such as unlimited drinks, music, wide range of drinks and associated them with a high frequency of risky behaviors due to the high consumption. Cash-bar places were referred to as relatively controlled consumption environment, in which consumption is limited by the amount of money available. The use of alcohol on the streets surrounding the university and drinking in get-together spaces were reported by a smaller number of participants, who considered this context as an environment for socializing with friends and classmates where alcohol is frequently present. Drinking in get-together places was reported only by students from public universities.

### Protective strategies

When participants were asked about effective self-care or protection strategies, they reported drinking slowly, consuming beverages with low alcohol content, short no-drinking intervals or alternating alcoholic beverages with water; eating before or during drinking, returning home in the company of friends, drinking in the presence of friends or trusted persons and inducing vomiting when feeling sick **(**Table 2**).**

**Table 2:** Examples of sentences on “Protective strategies” reported by participants

Categories	Examples		
Drinking slowly		“*One thing that takes your self-awareness away is drinking fast (…) Then, when you start feeling drunk, it comes in all at once and you get very sick*.” (21MSHL2-LR)	
Consuming low alcohol drinks		“At a party (…) If I drink three beers, I won’t get sick, but if I drink three Corotes®*, I will definitely get sick.” (19FFH1*-LR)*	
Stopping drinking, waiting for the drink “to go down” or alternate with drinking water.		*“when you feel like you’re getting drunk, take a break or control yourself, drink water in between drinks.“ (04FFH1-R)*	
Eating before or during the ingestion of drinks		“To *avoid getting sick, you know(…) you should drink a lot of water, not drink too much alcohol, eat a lot of food before you drink, and stuff like that.*” (03MFH1-LR)	
Returning home in the company of friend(s)		*“(…) going home drunk sometimes can be complicated, even if you are not driving, and you are just taking an Uber, you know, sometimes bad things could happen. It’s always good to have a friend around.” (01FIF1-HR)*	
Drinking in the company of friends or people you trust		*“(…) when you’re with your friends… you know everything is going to be okay. You trust those people. If you get drunk, there will be people to take care of you (…).”* (23FSH1-LR)	
Throwing up when you are feeling sick		*“Depending on the person, when they start feeling sick, sometimes they will prefer to throw up, you know, induce vomiting to try and feel better” (07MSH1-SD)*	

* low price popular drink with vodka (13.5% v/v – bottle 500 ml = 3 standard units)

Note: In order to maintain the confidentiality of the participants’ data, we created an alphanumeric code to identify the interviews, following the sequence: interview number (order of interviews), the gender of the respondent (“F” - female; “M” - male); the phase of college course they are in (“F” - first half of the course; “S” - second half of course), the general area of the course (“H” - humanities, “HL” - health, “E” – exact sciences) and the type of educational institution (“1” – public, “2” – private and the last part (after the hyphen) refers to their classification according to the AUDIT into: LR-Low risk, R- Risk or SD- Suggestive of dependence)

### Motivations for controlling alcohol consumption

When participants were asked about university students’ motivations to control alcohol consumption, they reported motivations related to themselves or others, which could be grouped into three factors: psychosocial, cognitive-behavioral, and self-monitoring (Table 3).

In the *p*s*ychosocial factors* category, we included issues related to the social systems in which the individual is inserted, mainly those related to religious issues, relationships with friends or family. The *cognitive-behavioral* category included perception, memories, self-belief, or other person’s beliefs, self-care, fear of judgment, a sense of commitment and responsibility, as well as bad memories of personal experiences. The *self-monitoring* category was related to feedback and behavior maintenance, occurring through self-perception of the physical and behavioral effects of alcohol, or through group dynamics, when friends warn them about their state of inebriation.

**Table 3:** Examples of sentences on “Motivations for consumption control” reported by the participants

Categories	Examples
Sense of commitment and responsibilities	*“(…) let’s face it, a hangover sucks (laughs). Waking up with a hangover at 9am on a Thursday to go to work (…) you’re going to be unproductive, you know. So, controlling your consumption is a responsibility.”* (15MSH1-R)
Memories of personal experiences	*“My control over alcohol consumption comes from having had problems in the past, blackouts, getting sick at parties (…) I’ve thrown up at bars, I’ve thrown up at a friend’s house (…).“* (06MSH1)
Self-perception of the effects of alcohol on your body and behavior	*“(…) I notice it because I try to focus on a fixed point and I automatically look up, that is, my eyes are on a fixed point, and without realizing it I look up. So, it feels really weird and that’s when I say ‘okay, time to stop”* (03MFH1-LR)
Dynamics of the group	“(“(…) S*ocial groups can (…) encouraging alcohol consumption* *or discouraging it* (…)” (06MSH1)
Financial and environmental issues	*“I think the person is also controlled by their budget, you are paying for each drink and you don’t want to spend too much money on it(…)”(07MSH1-SD)* *“There were people like, they get their first drinks but then they don’t get a second one, just because it was really hard to get it, since they had to wait in a huge queue, so they just go ‘oh no, never mind’ (…)”(*05MSE2-LR abstainer)
Self-care	*“I think that (self-care) might be one of the reasons (…). Like, why I don’t want to drink too much, because I want to drink enough so I am kind of tipsy and feel comfortable, but also want to be able to talk to people without making a fool of myself in front of them. But I don’t want to drink enough so I blackout or get sick (…)”.(*14FSH1-LR)
Fear of judgment	*“In addition to getting embarrassed, you know, because you’ll be with more mature people, right, you don’t want to play the fool and stuff like that,” (03MFH1-LR)*
Family issues	*“ (…) So, if the parents are strict, when their kids grow up and get their freedom, they go over the top. If the parents are more relaxed about it, then they tend not to exaggerate” (09FSHL2-LR)*
Religious issues	*“(I don’t drink too much) Because it goes against what I believe (I’m a Christian) and I believe that to get drunk is a sin. I’ve never been drunk, and it’s because I believe it’s not right (…) I don’t think drinking is wrong, so much so that I drink and stuff, I drink with my friends from work and stuff like that. (…) when I feel I start to get drunk, I stop drinking.”* (14FSH1-LR)

Note: In order to maintain the confidentiality of the participants’ data, we created an alphanumeric code to identify the interviews, following the sequence: interview number (order of interviews), the gender of the respondent (“F” - female; “M” - male); the phase of college course they are in (“F” - first half of the course; “S” - second half of course), the general area of the course (“H” - humanities, “HL” - health, “E” – exact sciences) and the type of educational institution (“1” – public, “2” – private and the last part (after the hyphen) refers to their classification according to the AUDIT into: LR-Low risk, R- Risk or SD- Suggestive of dependence)

### Influence of patterns of alcohol use and the environment

The use of protective strategies, motivation and sense of responsibility varied according to the student’s profile regarding patterns of drinking, and the drinking environment. Students with high alcohol consumption (AUDIT zones 2 and 3) and those with scores suggestive of dependence (AUDIT zone 4), reported low motivation to control alcohol consumption, frequency of use of protective strategies, and sense of responsibility. This situation is illustrated by the comment of one participant who said: “*The truth is that my friends are always the ones who take care of me*” (12MSHL2). Only those participants characterized as low or moderate alcohol users mentioned “responsibility” as a motivation to control the consumption.

The environment was also mentioned as a factor that influenced the motivation to control alcohol consumption and adopt protective strategies. Environments associated with high availability of alcohol, such as “open bar” parties, were also reported as triggers of high alcohol consumption, low motivation to control drinking or employment of protective strategies. The following comments exemplify this situation: *“I think that alcohol consumption changes according to the environment. If I’m at a party, I drink more than if I’m doing something more casual with friends in the afternoon, for example.”* (19FFH2*). “(…) I drink a lot (at open bar parties), first to make the ticket price worthwhile, second because “open bar” parties are something that do not happen every day (…) the best thing to do is drink like a crazy (…)”* (16MFH2).

Participants mentioned some motivations for controlling alcohol consumption: “(…) “*When I drink alcohol, you know, those social locks, they shrink, so like, one thing… when I got into college, I was very shy, and I didn’t go to bars (…) I started going, you know, and feeling comfortable in those environments because of alcohol, you know, interacting with people I don’t know, sometimes I end up using alcohol as a good-luck charm (…) and I believe most college students do it, too!* (…)” (06MSH1) “*Like I said, the motivation (to control alcohol consumption) both not to make a fool of myself in front of my friends, then motivation like for the money, the “great, I’m partying, I’m here, I paid for that, and if I get sick I will have to go home and I’m going to waste money” you know, I think it is more those two things!* (…)” (06MSH1).

The use of protective strategies was related to the context and the pattern of alcohol consumption “(…) in the Atlética* we already do that, you know, we have a party that is famous, “the Shower”, I don’t know if you’ve heard about it, then it’s an open bar party that lasts for ten, twelve hours on end, everybody knows the party because everybody gets sick, then there are moments when the open bar stops, then they give food, they give things with a high glycemic index for the people really hold on a little longer and go back a little sober” (09FSHL1).

*Atlética: Atlética is a university organization that aims to promote the social integration of students through sports.

## Discussion

The originality of this qualitative study on the main motivations for drinking, or not, and protective strategies employed by college students to control their alcohol consumption is the inclusion of participants with diverse levels of associated risks. Interviewing not only those classified as “at risk” or “suggestive of dependence” but also those with occasional, low consumption or currently abstainers brought new information on their behavior and influence of different contexts. The participants’ reports highlighted the importance of understanding the influence of both the context in which alcohol is used and peers’ behaviors. The students’ reports also allowed to explore their main motivations to drink, or not, and how they use protective strategies. These aspects are affected by individual (internal) and contextual (external) factors that interact in different ways, leading to higher or lower alcohol consumption and associated problems. Among the external factors that favor heavy use of alcohol, open bar parties and peer pressure stand out, while among the internal aspects factors such as low self-esteem and a desire to fit in emerge. These external and internal factors can contribute to a lower probability of using protective strategies in alcohol contexts. Overall, our findings are in line with those of Lorant at al (2013) [42]. According to them, students who were more exposed to some specific college environmental factors had a higher risk of abusive alcohol consumption. Most of these environmental factors were associated with social involvement, such as participation in student culture, pre-parties, and normative expectations.

A study carried out in the USA showed that engagement in protective behavior strategies (PBS) can increase or decrease depending on the celebration of a holiday, the time of year and the context of alcohol use [43]. According to Liden et al. (2014) [15], when motivations are positively linked with high alcohol consumption, people tend to have a lower frequency of PBS. On the other hand, when they are negatively related to alcohol consumption, PBS are used more often. In our study, participants had similar responses in the protective strategies category. Only participants characterized as low or moderate alcohol users, according to their AUDIT score, mentioned “responsibility” as a motivation to control consumption. The valuation of “responsibility” as a desirable characteristic of students may be a point which deserves attention in programs to promote PBS.

Previous studies carried out with Brazilian college students confirmed that university parties, especially open bar ones, were associated with high consumption of alcoholic beverages and associated risk behaviors [44]. They are considered by some students a leisure alternative and an escape valve from the pressure and anxiety arising from college life [44, 45, 20]. For these reasons, open bar parties and other similar environments deserve special attention in preventive and harm reduction actions [46].

On the other hand, some studies showed that most of college students, despite intense alcohol consumption in some specific contexts, in general, present a low-risk consumption pattern. [45, 47]. Most of them control their alcohol use, possibly by adopting protective strategies. Some of these behaviors seem to be spontaneously adopted, without previous preventive interventions, suggesting that individuals can develop protective strategies that reflect their values. Such findings contrast with the prohibitionist and “war on drugs” approaches, which do not offer youth credit in respect of their skills to act autonomously and consciously, and practice self-care in contexts where alcohol is present [46]. However, protective strategies can be influenced by the context of alcohol use, alcohol consumption patterns, and motivations to drink, or not to drink.

The psychosocial and cognitive-behavioral motivations observed in this study are aligned with the categories of external and internal motivators, previously described in other countries and cultures [17–19]. These studies describe external motivations as families, TV, and friends, and describe internal motivations as personal characteristics, curiosity, pleasure and shyness. Both the motivations for alcohol consumption and control are related to the individual, the context and “coping” behaviors, that is, the way they try to deal with external and/or internal demands [18, 19]. The self-monitoring category is related to the skill of observing internal and external signals, and adjusting the level of consumption based on bodily sensations and environmental perceptions. In this sense, the ability to self-monitor is closely related to self-regulation processes [27].

One of the main strengths of this study is its focus on the variables that may affect the students use of protective strategies for alcohol consumption. Through the qualitative analysis of the participants’ reports, it was possible to detect that the protective strategies can be influenced by contexts, motivations, and consumption patterns. These strategies were reported to be less used by people with a high consumption pattern, positive motivations for drinking and who were in environments that encourage the consumption of beverages, such as open bar parties. Therefore, understanding the factors that encourage drinking, the strategies that are already used by individuals to restrict their consumption of alcohol, and the patterns of consumption of the population is extremely important to develop more effective interventions.

The college students who participated in the study were mostly from middle-class and private institutions. To understand the phenomenon in other populations, further studies must be carried out in other samples with diverse cultural and socioeconomic characteristics. Regarding the level of risk associated with alcohol consumption, our sample had only one person classified as a “harmful user” and only a few men classified as “suggestive of dependence” users.

In summary, to develop preventive approaches and early interventions to reduce alcohol related problems among specific populations, it is crucial to have a comprehensive understanding of student´s motivations to drink, or not, as well the spontaneous strategies they employ to control their consumption. It is important to develop preventive programs considering the specificities of university alcohol consumption contexts, patterns of alcohol consumption, motivations for consuming and controlling alcohol ingestion strategies already existing among college students. Preventive programs in the university context can enhance the frequency of strategic protective behavior in this population, as well as interventional programs for self-care, body self-perception, and financial education. In this study students reported that saving money was one of the reasons for drinking less. Therefore, financial education seems to be an important factor to minimize consumption and its associated risks.

“Considering motivations and preventive strategies may vary according to the context and the pattern of use. As a result, they may impact targeting preventive damage reduction actions. In open bar parties, for example, the care should involve protective resources that do not depend on the decision-making of heavy drinkers. In this context, damage control actions such as wide availability of free water and food establish considerable protective relevance” [18, 19, 46].

## Conclusions

This study identified protective behavioral strategies and motivations to control drinking among Brazilian college students and found they were less used in contexts where there is an overall high alcohol consumption. These findings corroborate other recent studies in the area that point in the same direction [14, 15] and highlight the need for the development of preventive programs considering specific drinking contexts and patterns of alcohol consumption.

## Data Availability

The datasets used and/or analyzed during the current study are available from the corresponding author on reasonable request. Marina Noto Faria (marinanfaria@gmail.com).
